# Supplementary material for: Discordant Growth of Monozygotic Twins Starts at the Blastocyst Stage: A Case Study
Source: Stem Cell Reports. 2015 Nov 12;5(6):946–53. doi: 10.1016/j.stemcr.2015.10.006 (PMC4682124; doi:10.1016/j.stemcr.2015.10.006)
Supplement: Document S1. Figures S1–S4 [file mmc1.pdf]

Stem Cell Reports, Volume *5*

Supplemental Information

Discordant Growth of Monozygotic Twins Starts at the Blastocyst Stage: A Case Study

Laila Noli, Antonio Capalbo, Caroline Ogilvie, Yacoub Khalaf, and Dusko Ilic

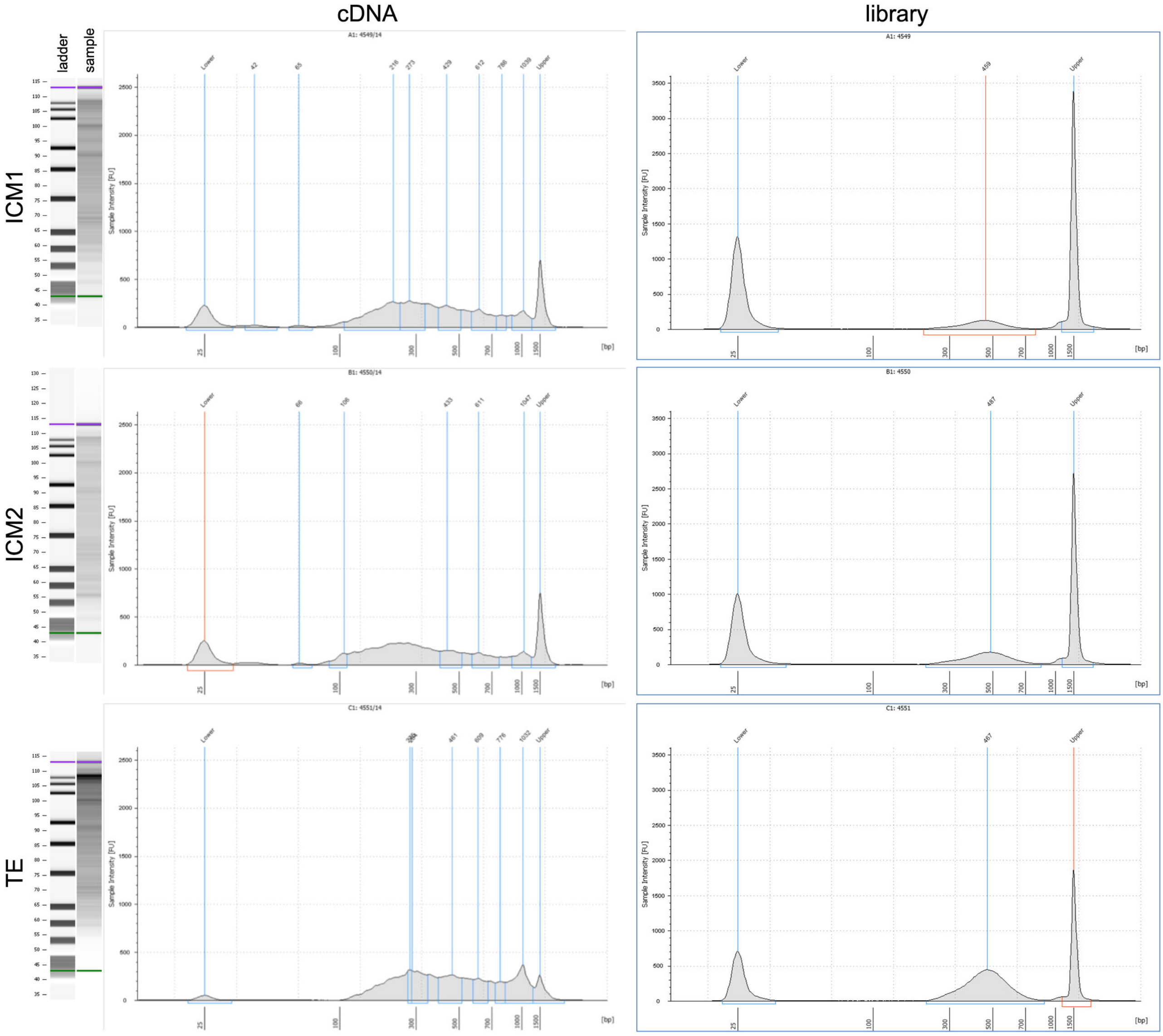

Figure S1. QC electropherograms. Size distribution profiles of cDNAs and libraries constructed from two ICM (ICM 1 and ICM2) and TE fraction of the monozygotic twin embryo.

A

Sample		Uniquely mapped reads	Properly paired
Illumina	ICM1	77.03%	74.32%
	ICM2	76.87%	74.53%
	TE	78.22%	76.76%
SOLiD	ICMP	80.64%	47.91%
	TEP	80.64%	48.40%

B

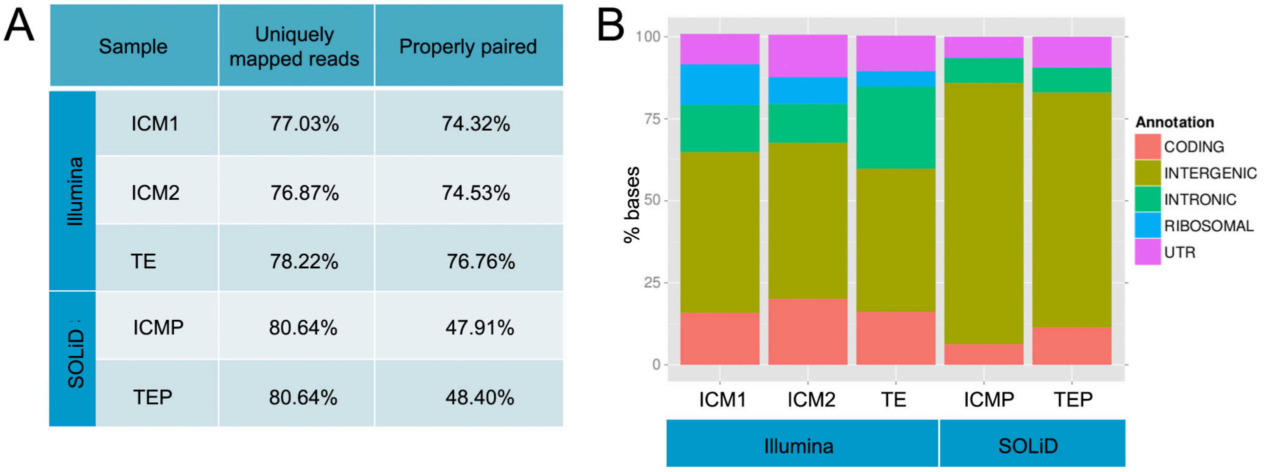

C

Sample		Ribosomal	Coding	UTR	Intronic	Intergenic	mRNA
Illumina	ICM1	12.35	15.89	9.26	14.42	48.96	25.15
	ICM2	8.25	20.08	12.90	11.82	47.60	32.98
	TE	4.74	16.13	10.76	25.02	43.70	26.89
SOLiD	ICMP	0.21	6.26	6.35	7.54	79.67	12.61
	TEP	0.16	11.40	9.32	7.53	71.61	20.72

D

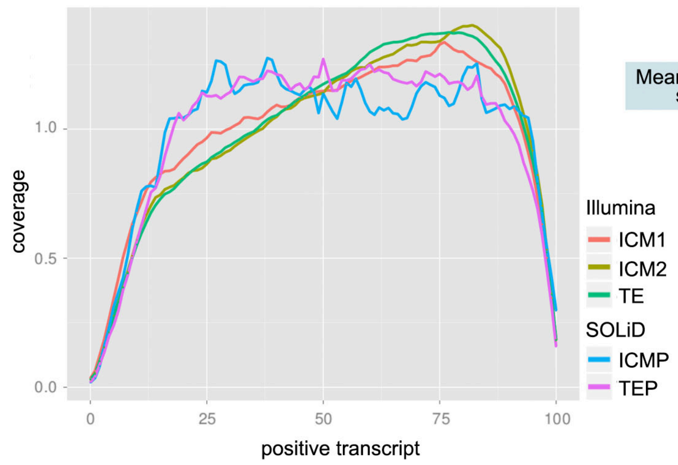

E

	Illumina			SOLiD	
	ICM1	ICM2	TE	ICMP	TEP
Mean insert size	67.9	79.4	72.5	40.7	44.5

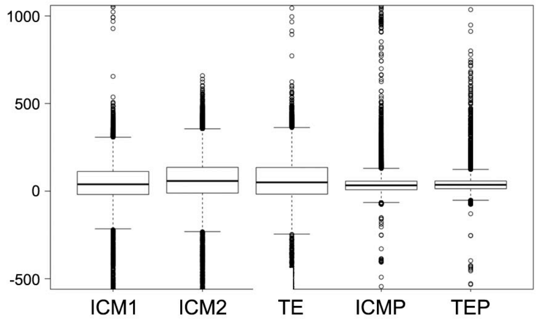

Figure S2. Quality control of mapping.

The samples are clustering by technology/batch rather than by biology. (A) Mapping statistics reveals high percentage of uniquely mapped reads on both platforms. Distribution of reads depending on genomic localization (B and C), the potential 5'/3' bias on transcript position (D), and estimated insert size (E) pointed to differences between two platforms.

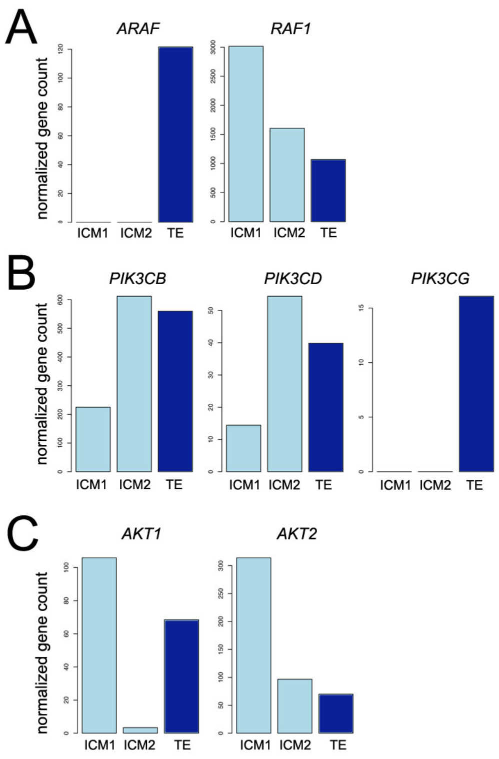

Figure S3. Expression levels of *RAF* (A), class I *PI3K* catalytic subunits (B) and *AKT* (C) gene family members that are not predominantly expressed in the ICM2.

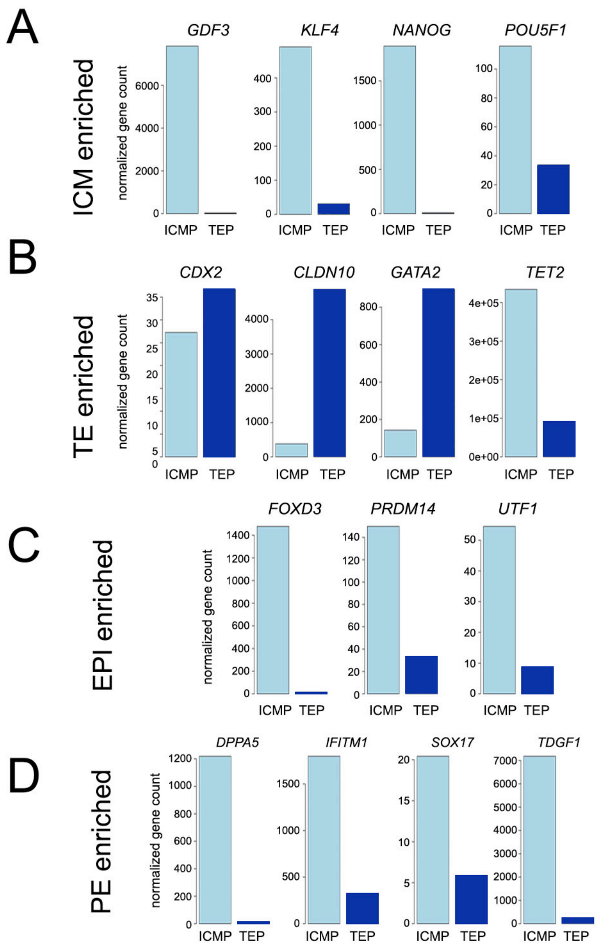

Figure S4. Lineage marker expression pattern confirms that the ICM samples are isolated form late blastocysts.

EPI, epiblast; ICM, inner cell mass; PE, primitive endoderm; TE, trophectoderm.
