# Supplementary material for: Discordant Growth of Monozygotic Twins Starts at the Blastocyst Stage: A Case Study
Source: Stem Cell Reports. 2015 Nov 12;5(6):946–53. doi: 10.1016/j.stemcr.2015.10.006 (PMC4682124; doi:10.1016/j.stemcr.2015.10.006)
Supplement: Document S2. Article plus Supplemental Information [file mmc2.pdf]

Stem Cell Reports
Report
OPEN ACCESS

Discordant Growth of Monozygotic Twins Starts at the Blastocyst Stage: A Case Study

Laila Noli,[1] Antonio Capalbo,[2,3] Caroline Ogilvie,[4] Yacoub Khalaf,[1] and Dusko Ilic[1,*]
[1]Division of Women's Health, Faculty of Life Sciences and Medicine, King's College London and Assisted Conception Unit, Guy's Hospital, London SE1 9RT, UK
[2]GENERA: Centre for Reproductive Medicine, Clinica Valle Giulia, 00197 Rome, Italy
[3]GENETYX: Molecular Genetics Laboratory, Via Fermi 1, 36063 Marostica, Italy
[4]Genetics Laboratories, Guy's Hospital, SE1 9RT London, UK
*Correspondence: dusko.ilic@kcl.ac.uk
http://dx.doi.org/10.1016/j.stemcr.2015.10.006
This is an open access article under the CC BY license (http://creativecommons.org/licenses/by/4.0/).

SUMMARY

Discordant growth is a common complication of monochorionic/diamniotic pregnancies; in approximately 50% of cases, the cause is unknown. The case presented here suggests that discordant growth of monozygotic twins could start during preimplantation development. Two inner cell masses (ICMs) within the same blastocyst may originate in uneven splitting of a single "parental" ICM, or the two ICMs may be formed independently de novo. We studied the transcriptomes of two morphologically distinct ICMs within a single blastocyst using high-resolution RNA sequencing. The data indicated that the two ICM were at different stages of development; one was in the earliest stages of lineage commitment, while the other had already differentiated into epiblast and primitive endoderm. IGF1-mediated signaling is likely to play a key role in ICM growth and to be the major driver behind these differences.

INTRODUCTION

Monozygotic (MZ) twins originate from the same conception event and are genetically identical. Approximately 80% of MZ twins originate from monochorionic/diamniotic pregnancies, meaning that they share the placenta and chorion, whereas each twin has its own amnion. These monochorionic/diamniotic MZ twins form if the inner cell mass (ICM), the part of the embryo that will give rise to the fetal body, splits at the early blastocyst stage, 4–6 days post-fertilization. Molecular events leading to this splitting are still in the domain of speculation. Time-lapse cinematography suggests that, at least in vitro, the reason might be purely mechanical; twins have been shown to form after blastocyst collapse and re-expansion, splitting the ICM into two groups of cells (Payne et al., 2007).

It is well documented that the incidence of MZ twins is higher following assisted reproduction techniques (Blickstein et al., 2003; Vitthala et al., 2009; Knopman et al., 2014; Tocino et al., 2015); however, we have rarely seen a good-quality blastocyst with two distinct ICMs among embryos donated for research in our center. High-resolution single-cell transcriptome analysis of the temporal and spatial patterns of gene expression in the human preimplantation embryos has been reported (Xue et al., 2013; Yan et al., 2013), and when we recently encountered such an embryo, we took the opportunity to compare the ICMs at the transcriptome level using next-generation sequencing (NGS).

RESULTS

The expanded day 6 blastocyst displayed two distinct ICMs 4 hr post-thawing, (Figure 1A). Single cells and cell borders were clearly visible in ICM1, whereas the cells in ICM2 were more tightly packed and no single cell could be distinguished, indicating that ICM2 is either better quality or more advanced than ICM1. To further explore this morphological discrepancy, we separated ICM1 and ICM2 from trophectoderm (TE) and by NGS evaluated the transcriptome of each of the three fractions. Tiered quality-control (QC) steps suggested good quality of cDNA and the library (Figure S1), as well as high homogeneity between samples (Figure S2). Nevertheless, we are aware that comparing two conditions with no replicates is not ideal and strong events and false positives may be missed.

Unexpectedly, the ICM1 and ICM2 transcriptome profiles did not cluster together; the ICM1 transcriptome was more similar to TE than to ICM2 (Figures 1B–1E). We found that ICM1 and ICM2 have 842 differently regulated genes with a fold change ≥ 2 and p value ≤ 0.5, whereas the difference between ICM1 and TE was only in 128 genes and ICM2 and TE differed in expression of 356 genes. Pathway analyses drew our attention to insulin-like growth factor 1 (IGF1) signaling (Figures 2A and 2B). *IGF1* expression was relatively high in ICM2 and nearly undetectable in ICM1 (Figure 2C).

IGF1 acts through multiple pathways, including RAS/RAF/MAPK and PI3K/AKT. We therefore investigated whether the expression levels of downstream mediators,

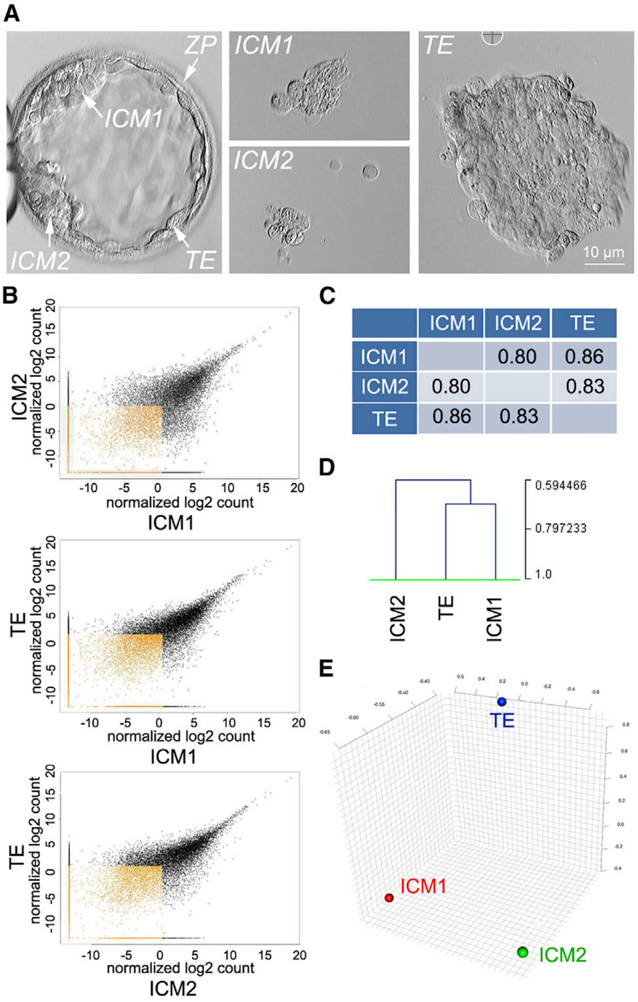

Figure 1. Transcriptome of Two ICMs of the MZ Twin Embryo Does Not Cluster Together

(A) Two ICMs (ICM1 and ICM2) of a day 6 natural MZ twin embryo mechanically separated from TE.

(B–E) Sample clustering used correlation of normalized read counts of expressed genes and average linkage clustering. Comparisons of normalized gene counts (B), correlation (C), clustering based on correlation (D), and principal component analysis (E) demonstrate that ICM1 is closer to TE than to ICM2.

members of RAF, PI3K, and AKT families, are different between ICM1 and ICM2 (Figures 2C and S3). Interestingly, from three RAF family members, *BRAF* was specifically expressed in ICM2, whereas *ARAF* was expressed only in TE. *RAF1* was found in all three fractions, at a level twice as high in ICM1 as in ICM2. Among class I PI3K catalytic subunits, only *PIK3CA* was expressed almost exclusively in ICM2. *PIK3CB* and *PIK3CD* were expressed in all three fractions, whereas *PIK3CG* was only in TE. Expression levels of AKT family members were also cell fraction specific: *AKT3* was predominantly expressed in ICM2, whereas *AKT1* was expressed in ICM1 and TE and was barely detectable in ICM2. *AKT2* was expressed in all three fractions but at a higher level in ICM1 than in ICM2. Although we do not know whether the protein expression parallels mRNA levels and, moreover, whether the signaling cascade is active, the data suggest that ICM1 is deficient for IGF1-mediated signaling. These data may indicate that the IGF1 pathway in the ICM of the human preimplantation embryos is linked to specific members of the RAF, PI3K, and AKT families.

The differences between ICM1 and ICM2 in global gene expression and particularly in expression of IGF1 pathway members suggested that they are distinct not only morphologically (Figure 1A) but also on a molecular level. Next, we examined relative expression levels of ICM and TE markers (Yan et al., 2013). ICM markers *GDF3*, *KLF4*, and *POU5F1* were higher in ICM2, whereas *NANOG* was higher in ICM1 (Figure 3A). TE markers showed a more interesting pattern (Figure 3B); although all were expressed in TE, only *CDX2* was TE specific. *CLDN10* and *GATA2* were expressed around the same level in ICM1 as in TE, whereas they were barely detectable in ICM2. *TET2* was expressed in all three fractions, the highest in TE and the lowest in ICM2. Expression of TE markers in ICM1 and not in ICM2 raises the possibility of cross-contamination of the ICM1 fraction with TE cells during the isolation process. Although we cannot exclude this, a contamination with TE cells is highly unlikely—CDX2 expression in ICM1 would parallel a pattern of other TE markers, and it does not. In contrast to in the mouse, in the human CDX2 is first detected in day 5 human embryos and is exclusively expressed in TE (Niakan and Eggan, 2013). Therefore, together with the data from the IGF1 pathway, these results strongly indicate that ICM1 and ICM2 were at different stages of development.

The first cell fate decision occurs during the transition from morula to blastocyst. The allocation of cells to the ICM occurs through cell divisions in which outside cells divide in an orientation that directs one daughter inward (Johnson and Ziomek, 1981). The ICM has been shown not to be a homogenous group of cells (Chazaud et al., 2006), and it is to be expected that at the initial stages of segregation, some of the very early ICM cells retain detectable levels of expression of the proteins present in parental cells. In an early day 5 blastocyst with a small but morphologically distinct ICM, we were able to detect within the ICM the cells expressing NANOG and GATA2 (Figure 3C). To explore further the possibility that ICM1 is at a stage of development earlier than that of ICM2, we investigated the expression pattern of *YAP1* (Figure 3D). YAP1 can shuttle between the cytoplasm and the nucleus, and its nuclear localization is linked to an active role in the TAED4-mediated induction of *CDX2* expression (Lorthongpanich

A KEGG Pathway Analysis on Regulated Genes

Significant Pathways (6)

Pathway Description (KEGG)	Nb Genes in Pathway	Nb Regulated Genes (Up / Down)	P-Value (All)	P-Value (Up)	P-Value (Down)	Min P-Value
Glioma	63	9 (4/5)	4.16E-03	NA	2.47E-02	4.16E-03
N-Glycan biosynthesis	46	6 (6/0)	4.04E-02	4.25E-03	NA	4.25E-03
ErbB signaling pathway	87	10 (6/4)	9.49E-03	NA	NA	9.49E-03
Progesterone-mediated oocyte maturation	86	6 (0/6)	NA	NA	1.75E-02	1.75E-02
Prostate cancer	89	9 (4/5)	3.04E-02	NA	NA	3.04E-02
Amino sugar and nucleotide sugar metabolisr	44	6 (3/3)	3.42E-02	NA	NA	3.42E-02

Pathway Description (KEGG)	FAST DB STABLE ID	Gene Symbol	Regulation	Fold-Change	P-Value
Glioma	GSHG0017610	CALM2	down	6	NA
	GSHG0009351	SOS2	up	inf	NA
	GSHG0028642	BRAF	down	93	NA
	GSHG0010191	IGF1R	up	inf	3.00E+00
	GSHG0013057	TP53	up	79	4.00E+00
	GSHG0025452	E2F3	down	inf	4.00E+00
	GSHG0007722	IGF1	down	inf	4.00E+00
	GSHG0021342	PIK3CA	down	77	5.00E+00
	GSHG0012740	PRKCA	up	471	NA
N-Glycan biosynthesis	GSHG0022279	ALG3	up	inf	3.00E+00
	GSHG0000584	ALG6	up	88	3.00E+00
	GSHG0005837	GANAB	up	105	3.00E+00
	GSHG0021775	RFT1	up	inf	NA
	GSHG0031755	ALG13	up	19	3.00E+00
	GSHG0010922	NAGPA-AS1	up	inf	2.00E+00
ErbB signaling pathway	GSHG0002768	ABL2	up	inf	2.00E+00
	GSHG0009351	SOS2	up	inf	NA
	GSHG0028642	BRAF	down	93	NA
	GSHG0016739	NCK2	up	914	NA
	GSHG0021342	PIK3CA	down	77	5.00E+00
	GSHG0013418	STAT5B	up	inf	3.00E+00
	GSHG0006740	ERBB3	up	214	4.00E+00
	GSHG0012740	PRKCA	up	471	NA
	GSHG0032069	ELK1	down	672	NA
	GSHG0014903	PAK4	down	147	2.00E+00
Progesterone-mediated oocyte maturation	GSHG0028642	BRAF	down	93	NA
	GSHG0009633	HSP90AA1	down	4	NA
	GSHG0013509	CDC27	down	31	NA
	GSHG0032011	RPS6KA3	down	44	4.00E+00
	GSHG0007722	IGF1	down	inf	4.00E+00
	GSHG0021342	PIK3CA	down	77	5.00E+00
Prostate cancer	GSHG0009351	SOS2	up	inf	NA
	GSHG0028642	BRAF	down	93	NA
	GSHG0008441	LINC00598	up	inf	3.00E+00
	GSHG0010191	IGF1R	up	inf	3.00E+00
	GSHG0013057	TP53	up	79	4.00E+00
	GSHG0025452	E2F3	down	inf	4.00E+00
	GSHG0007722	IGF1	down	inf	4.00E+00
	GSHG0021342	PIK3CA	down	77	5.00E+00
	GSHG0009633	HSP90AA1	down	4	NA
Amino sugar and nucleotide sugar metabolism	GSHG0020429	PMM1	up	inf	2.00E+00
	GSHG0017697	GFPT1	down	42	3.00E+00
	GSHG0006534	CMAS	up	62	3.00E+00
	GSHG0016547	NAGK	up	966	2.00E+00
	GSHG0023207	UGDH	down	inf	NA
	GSHG0001855	GALE	down	inf	3.00E+00

B REACTOME Pathway Analysis on Regulated Genes

Significant Pathways (4)

Pathway Description (REACTOME)	Nb Genes in Pathway	Nb Regulated Genes (Up / Down)	P-Value (All)	P-Value (Up)	P-Value (Down)	Min P-Value
Metabolism of lipids and lipoproteins	150	8 (8/0)	NA	2.67E-02	NA	2.67E-02
Hemostasis	235	15 (9/6)	3.31E-02	NA	NA	3.31E-02
Signaling by BMP	23	4 (2/2)	4.45E-02	NA	NA	4.45E-02
APC-Cdc20 mediated degradation of Nek2A	23	3 (0/3)	NA	NA	4.57E-02	4.57E-02

Pathway Description (REACTOME)	FAST DB STABLE ID	Gene Symbol	Regulation	Fold-Change	P-Value
Metabolism of lipids and lipoproteins	GSHG0029241	SQLE	up	21	2.00E+00
	GSHG0024764	HMGCS1	up	106	NA
	GSHG0029948	DGAT1	up	564	4.00E+00
	GSHG0017455	SDC1	up	inf	5.00E+00
	GSHG0002135	PPAP2B	up	286	3.00E+00
	GSHG0016924	GPD2	up	17	4.00E+00
	GSHG0020742	ABHD5	up	236	4.00E+00
	GSHG0016311	HADHB	up	678	NA
Hemostasis	GSHG0017610	CALM2	down	6	NA
	GSHG0004845	SERPING1	down	1820	2.00E+00
	GSHG0025682	BRPF3	up	inf	2.00E+00
	GSHG0017288	INPP5D	up	inf	4.00E+00
	GSHG0011134	ITGAM	down	inf	NA
	GSHG0021913	CD47	up	inf	NA
	GSHG0021342	PIK3CA	down	77	5.00E+00
	GSHG0007722	IGF1	down	inf	4.00E+00
	GSHG0002367	SLC16A1	up	55	NA
	GSHG0007526	ITGA5	up	inf	NA
	GSHG0031987	PIR	up	358	NA
	GSHG0012740	PRKCA	up	471	NA
	GSHG0024046	F2R	down	inf	3.00E+00
	GSHG0009242	SLC7A8	up	24	NA
	GSHG0004954	PLCB3	up	inf	4.00E+00
Signaling by BMP	GSHG0018555	BMP2	down	18	NA
	GSHG0016905	ACVR2A	up	inf	2.00E+00
	GSHG0020706	ACVR2B	down	1766	2.00E+00
	GSHG0003523	BMPR1A	up	74	4.00E+00
APC-Cdc20 mediated degradation of Nek2A	GSHG0013509	CDC27	down	31	NA
	GSHG0018789	UBE2C	down	19	NA
	GSHG0000446	CDC20	down	33	4.00E+00

C

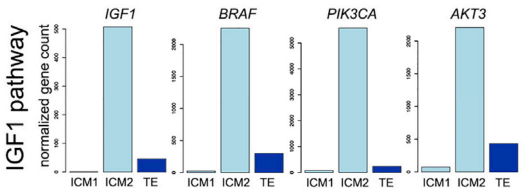

Figure 2. Pathway Analysis of Genes Differently Expressed between ICM1 and ICM2 Highlighted the IGF1 Signaling Pathway

(A) Kyoto Encyclopedia of Genes and Genomes database pathway analysis on regulated genes highlighted six pathways. Three of the six pathways involve the IGF1/BRAF/PIK3CA signaling cascade.

(B) Reactome database pathway analysis on regulated genes highlighted four pathways, from which one involves the IGF1/PIK3CA signaling cascade.

(C) Expression of *IGF1* and downstream mediators *BRAF*, *AKT3*, and *PIK3CA* of its signaling pathway is higher in ICM2 than in ICM1 or TE.

et al., 2013; Home et al., 2012; Nishioka et al., 2009). We found that ICM1 was expressing higher levels of *YAP1* than those of not only ICM2 but also TE, which was somewhat discordant with the published mouse data. Therefore, we investigated the YAP1 expression pattern in morula to late blastocyst stages of human embryo development. In contrast to the mouse, we found that YAP1 is heavily expressed and localized in the nuclei of ICM cells in the early stage blastocyst, whereas at later stages, similarly to the mouse, the expression in the ICM is exclusively cytoplasmic and seems to be dwindling in both the ICM and most TE cells. Our immunostaining data support the hypothesis that ICM1 and ICM2 are at different stages of the development, with ICM2 being developmentally more advanced than ICM1.

To test this further, we looked into the expression of epiblast (Epi) and primitive endoderm (PE) markers (Yan et al., 2013), which would be expressed only in an advanced ICM. The Epi markers *FOXD3*, *PRDM14*, and *UTF1* were found almost exclusively in ICM2. All PE markers were found in both ICMs, although their expression was higher in ICM2, especially *DPPA5* and *IFITM1* (Figure 3E).

If BRAF, PIK3CA, and AKT3 are specific for IGF1 downstream signaling at that stage of development, as the data from the ICM2 sample suggested (Figure 4A), their expression would show a similar pattern in other embryos. We pooled together the same type of samples: ICM pool (ICMP; n = 3) and TE pool (TEP; n = 3) from three late blastocysts and analyzed them using a different RNA sequencing (RNA-seq) platform (the SOLiD platform from Life Technologies instead of Illumina). We understand that the data obtained from two different platforms are not directly comparable. However, the data obtained should reflect the biology of the samples, and if the BRAF/PIK3CA/AKT3 signaling pathway or pathways are specific for ICM, their expression should show a similar trend regardless of the RNA-seq platform used. The data from the SOLiD platform suggested that may not be the case (Figures 4B–4F and S4). None of RAF and AKT family members showed strong ICM specificity as detected in

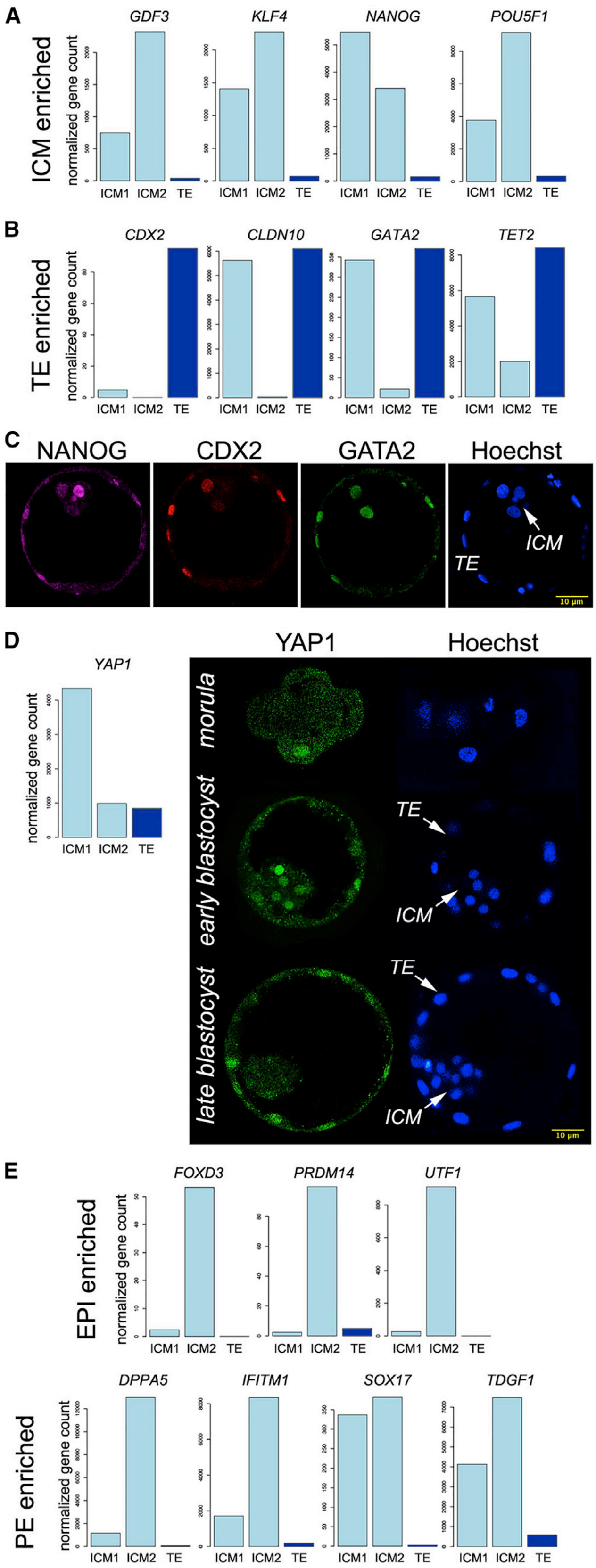

Figure 3. Lineage Marker Expression Pattern Suggests that ICM2 Is Developmentally More Advanced Than ICM1

(A) Genes known to have significantly higher expression in ICM (*GDF3*, *KLF4*, *NANOG*, and *POU5F1*) show the expected pattern.

(B) Among four genes known to have significantly higher expression in TE (*CDX2*, *CLDN10*, *GATA2*, and *TET2*), two of them, *CLDN10* and *GATA2*, are equally high expressed in ICM1 and in TE.

(C) At the early blastocyst stage, some cells still have dual expression of ICM and TE markers. A confocal image shows a day 5 blastocyst immunostained for the ICM marker NANOG and TE markers CDX2 and GATA2.

(D) Expression of transcription factor *YAP1*, which plays a role in initiating *CDX2* expression and committing cells to the TE lineage, is higher in ICM1 than in ICM2. YAP1 immunostaining of preimplantation embryos of different stages of development shows that YAP1 is detected in the nuclei of ICM cells only at the early stage of blastocyst formation. At the late blastocyst stage, YAP1 nuclear localization is restricted to TE.

(E) Expression of Epi and PE markers is higher in ICM2 than in ICM1, suggesting that ICM2 is developmentally more advanced than ICM1.

ICM2. In the newly analyzed samples, PIK3CD and PIK3CG seemed to be more specific to ICM than was PIK3CA, which stood out from ICM2 analyses.

DISCUSSION

MZ twins are recognized to be at greater risk of congenital anomalies and other morbidity than dizygotic ones (Lewi et al., 2008). One of the most common complications is severe discordant growth, which is defined as 25% or greater difference in birth weight for live-born twins or cases resulting in intrauterine fetal death and 25% or greater difference in estimated fetal weight in the absence of twin-to-twin transfusion syndrome. Such severe discordant growth has been reported in 7%–14% of all monochorionic/diamniotic pregnancies (Harper et al., 2013; Lewi et al., 2008; Acosta-Rojas et al., 2007; Fick et al., 2006). Hidden loss of such pregnancies is not known. Unequal placental sharing, defined as a placental territory discordance of ≥ 1.5 or as one twin receiving blood from >60% of the placenta, can explain about 50% of pregnancies with discordant growth; the cause of the other half in most cases is unknown (Lewi et al., 2007; Fick et al., 2006).

NGS and immunostaining data in this study suggest that discordant growth of MZ twins might start as early as the blastocyst stage (Figure 4A). Tocino et al. (2015) reported a case of asymmetric MZ twinning after a single embryo transfer. Discordant growth was observed by ultrasound as early as 24 days following the embryo transfer, and a difference in size was constant throughout the pregnancy. The prevalence of this early stage discordant growth is

A

Markers			ICM1	ICM2	TE
ICM-enriched		*GDF3*	+	+++	–
		KLF4	++	+++	–
		NANOG	+++	++	–
		POU5F1	+	+++	–
TE-enriched		*CDX2*	–	–	+++
		CLDN10	+++	–	++
		GATA2	+++	–	+++
		TET2	++	+	+++
		YAP1	+++	+	+
EPI-enriched		*FOXD3*	–	+++	–
		PRDM14	–	+++	–
		UTF1	–	+++	–
PE-enriched		*DPPA5*	+	+++	–
		IFITM1	+	+++	–
		SOX17	+++	+++	–
		TDGF1	++	+++	–
IGF1-mediated signaling	*IGF1*		–	+++	+
	RAF	*ARAF*	–	–	+++
		BRAF	–	+++	+
		RAF1	+++	++	+
	PIK3C	*PIK3CA*	–	+++	–
		PIK3CB	+	+++	+++
		PIK3CD	+	+++	++
		PIK3CG	–	–	+++
	AKT	*AKT1*	+++	–	++
		AKT2	+++	+	+
		AKT3	–	+++	+

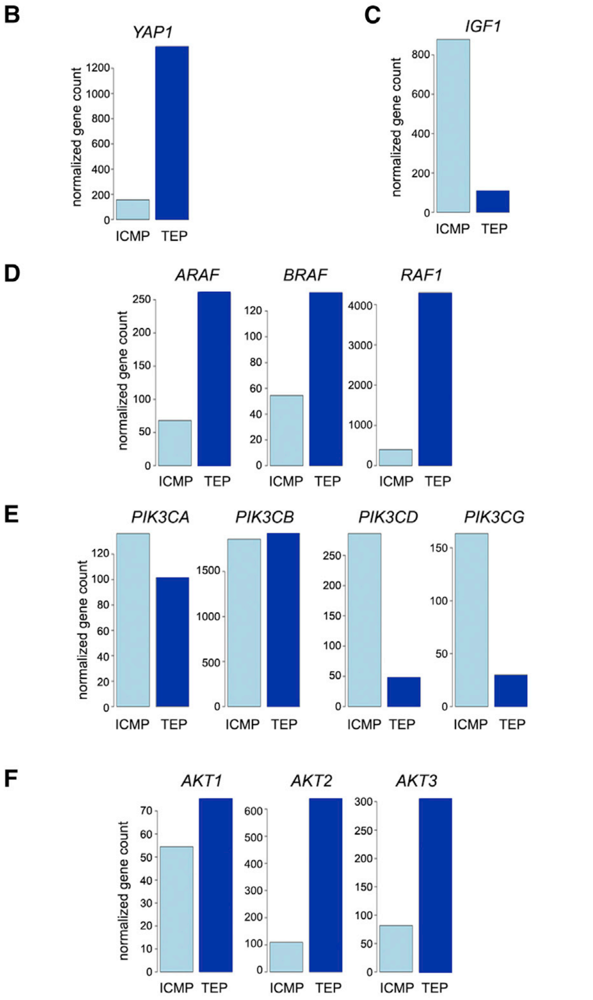

Figure 4. BRAF/PIK3CA/AKT3 Signaling Is Not Specific for the ICM of Late Blastocysts

(A) Gene expression pattern of lineage markers and IGF1-mediated signaling pathway is shown in three fractions of the MZ twin blastocyst, ICM1, ICM2, and TE.

(B) High *YAP1* expression levels in TEP (n = 3) and low levels in ICMP (n = 3) confirm that the samples used for these analyses are isolated from the late stage blastocysts.

(C–F) Although IGF1 expression is high in ICMP (C), downstream mediators BRAF (D), PIK3CA (E), and AKT3 (F), exclusively expressed in ICM2 (A), do not show an ICM-specific pattern in ICMP (n = 3).

not known, and further case reports and studies are needed. Interestingly, the couple who donated the embryo for this study had been through multiple unsuccessful in vitro fertilization cycles, and an inherited pathology as causative of the discordant development observed remains as a distant possibility.

Our studies suggest that the role of IGF1-mediated signaling in ICM growth is worthy of further exploration and may shed light on molecular pathways controlling early human embryo development. IGF1, also known as somatomedin C, induces growth and metabolism of preimplantation embryos in vitro (Harvey and Kaye, 1992). Studies in multiple species have demonstrated that IGF1 selectively stimulates the proliferation of cells in an ICM, as well as protecting them from apoptosis (Green and Day, 2013; Thieme et al., 2012; Bonilla et al., 2011; Velazquez et al., 2009; Kimber et al.; 2008; Spanos et al., 2000; Lighten et al., 1998). Recent reports have correlated levels of IFG1 in follicular fluid with both quality of embryos and clinical pregnancy during in vitro fertilization cycles (Mehta et al., 2013). However, preimplantation embryos exposed to high non-physiological concentrations of IGF1 undergo extensive apoptosis of the ICM nuclei (Chi et al., 2000; Makarevich and Markkula, 2002; Velazquez et al., 2011), suggesting that IGF1 signaling in preimplantation embryos is tightly regulated. In light of these reports, including our findings, the possibility of adding IGF1 into embryo culture media during assisted reproduction to improve the odds for better-quality embryos and fetal growth post-transfer should be explored further, and downstream pathways and molecular mechanisms should be investigated.

The different stages of development of the two ICMs might result from an uneven split of an initial single ICM during blastocyst collapse. A delay in formation of intercellular adhesions within the ICM might make the ICM prone to splitting (Togashi et al., 2015). The smaller part, in this case ICM1, might be lagging behind due to a lack of critical cell mass. However, we cannot exclude the possibility that ICM1 and ICM2 formed independently de novo at

different time points (Noli et al., 2015). TE cells from the human non-expanded blastocysts are not fully committed; isolated and reaggregated TE cells are able to develop into blastocysts with ICM cells expressing NANOG (De Paepe et al., 2013). Either way, our case report suggests new avenues to studies of cellular and molecular events behind MZ twinning and discordant growth, commonly associated with monochorionic/diamniotic pregnancies, as well as lineage commitment at the earliest stages of human development.

EXPERIMENTAL PROCEDURES

The work described here was done under license from the UK Human Fertilization and Embryology Authority (research license R0075) and has local ethical approval (UK National Health Service Research Ethics Committee, reference 06/Q0702/90). Informed consent was obtained from all subjects, and the experiments conformed to the principles set out in the WMA Declaration of Helsinki and the NIH Belmont Report. No financial inducements were offered for donation.

Human Embryo Culture

The embryos were thawed using Quinn's Advantage thaw kit according to the manufacturer's instructions (Sage). After thawing, the embryos were cultured in 40-μl microdrops of Quinn's blastocyst medium supplemented with 10% synthetic protein serum substitute (Sage) under mineral oil (Sage) at 37°C in 5% CO_2 and 5% O_2 in a humidified atmosphere. ICM and TE were separated mechanically as described (Capalbo et al., 2013).

RNA-Seq and Bioinformatics

TE and ICM fractions of the MZ twin embryo were lysed in 1 μl of lysis buffer A from the Prelude direct lysis module (NuGEN), and cDNA was synthesized and amplified using the Ovation RNA-seq system V2 enabled by proprietary ribo-single primer isothermal amplification technology following the manufacturer's instructions (NuGEN). The cDNA was purified using the MinElute reaction cleanup kit (QIAGEN), and it was analyzed using the 2100 Bioanalyzer with the DNA 1000 LabChip and the 2200 TapeStation (Agilent). Unidirectional strand-specific 450–500 bp libraries were constructed on the Apollo 324 NGS system (IntergenX) and then sequenced on the HiSeq 2000 system (Illumina) by Oxford Gene Technology. The libraries were quantified using Qubit (Life Technologies), and the size profile was analyzed on the 2200 TapeStation (Agilent). Reads were aligned to the human genome (hg19) using Spliced Transcripts Alignment to a Reference v.2.4.0 (Dobin et al., 2013).

The cDNA was prepared from TE and ICM fractions pooled from three normal embryos using the same protocol. The library construction and the NGS on SOLiD platform (Life Technologies) were performed by Microsynth, and the alignments to hg19 were performed using LifeScope v.2.5 software (http://www.thermofisher.com/uk/en/home/technical-resources/software-downloads/lifescope-genomic-analysis-software.html).

GenoSplice Technology performed QC, processing, and further analyses of the data. For each gene present in the Friendly Alternative Splicing and Transcripts Database v.2014_2, reads aligning on constitutive regions (that are not prone to alternative splicing) were counted. Based on these read counts, normalization and differential gene expression were performed using DESeq (v.1.16.0 on R v.3.1.3) (Anders and Huber, 2010). Sample clustering was performed with MeV 4.9 (Saeed et al., 2003) using correlation of normalized read counts of expressed genes and average linkage clustering.

Immunostaining

Immunostaining was performed as described (Niakan and Eggan, 2013). Primary antibodies were rabbit polyclonal anti-YAP1 (Cell Signaling Technology, Catalog No. 4912) and anti-GATA2 (Santa Cruz Biotechnology, catalog no. SC-9008), goat polyclonal anti-NANOG (R&D Systems, catalog no. AF1997), and mouse monoclonal anti-CDX2 (BioGenex, catalog no. MU392A-UC). Secondary antibodies were purchased from Life Technologies. Nuclei are visualized with DAPI (Vector Laboratories). Confocal microscopy was carrying out using a Leica TCS SP5 microscope. Images were process using ImageJ64 and Adobe Photoshop Creative Cloud 2014 software.

ACCESSION NUMBERS

The accession number for the RNA-seq data reported in this paper is Sequence Read Archive: SRP063754.

SUPPLEMENTAL INFORMATION

Supplemental Information includes four figures and can be found with this article online at http://dx.doi.org/10.1016/j.stemcr.2015.10.006.

AUTHOR CONTRIBUTIONS

L.N. and A.C. performed experiments. L.N., C.O., Y.K., and D.I. contributed to the design of the study and took part in the analysis of the results and writing the manuscript.

ACKNOWLEDGMENTS

This project was supported by the Saudi Arabian Government studentship to L.N. and Y.K.'s and D.I.'s incentive funds. We thank to N. Fogarty and K. Niakan from the Division of Stem Cell Biology and Developmental Genetics, National Institute for Medical Research, Mill Hill, for technical help and discussion. We thank the staff at the Assisted Conception Unit of Guy's and St Thomas' NHS Foundation Trust for supporting the research program. We are especially indebted to the patients who donated embryos.

Received: May 24, 2015
Revised: October 8, 2015
Accepted: October 8, 2015
Published: November 12, 2015

REFERENCES

Acosta-Rojas, R., Becker, J., Munoz-Abellana, B., Ruiz, C., Carreras, E., and Gratacos, E.; Catalunya and Balears Monochorionic

Network (2007). Twin chorionicity and the risk of adverse perinatal outcome. Int. J. Gynaecol. Obstet. *96*, 98–102.

Anders, S., and Huber, W. (2010). Differential expression analysis for sequence count data. Genome Biol. *11*, R106.

Blickstein, I., Jones, C., and Keith, L.G. (2003). Zygotic-splitting rates after single-embryo transfers in in vitro fertilization. N. Engl. J. Med. *348*, 2366–2367.

Bonilla, A.Q., Ozawa, M., and Hansen, P.J. (2011). Timing and dependence upon mitogen-activated protein kinase signaling for pro-developmental actions of insulin-like growth factor 1 on the preimplantation bovine embryo. Growth Horm. IGF Res. *21*, 107–111.

Capalbo, A., Wright, G., Elliott, T., Ubaldi, F.M., Rienzi, L., and Nagy, Z.P. (2013). FISH reanalysis of inner cell mass and trophectoderm samples of previously array-CGH screened blastocysts shows high accuracy of diagnosis and no major diagnostic impact of mosaicism at the blastocyst stage. Hum. Reprod. *28*, 2298–2307.

Chazaud, C., Yamanaka, Y., Pawson, T., and Rossant, J. (2006). Early lineage segregation between epiblast and primitive endoderm in mouse blastocysts through the Grb2-MAPK pathway. Dev. Cell *10*, 615–624.

Chi, M.M., Schlein, A.L., and Moley, K.H. (2000). High insulin-like growth factor 1 (IGF-1) and insulin concentrations trigger apoptosis in the mouse blastocyst via down-regulation of the IGF-1 receptor. Endocrinology *141*, 4784–4792.

De Paepe, C., Cauffman, G., Verloes, A., Sterckx, J., Devroey, P., Tournaye, H., Liebaers, I., and Van de Velde, H. (2013). Human trophectoderm cells are not yet committed. Hum. Reprod. *28*, 740–749.

Dobin, A., Davis, C.A., Schlesinger, F., Drenkow, J., Zaleski, C., Jha, S., Batut, P., Chaisson, M., and Gingeras, T.R. (2013). STAR: ultrafast universal RNA-seq aligner. Bioinformatics *29*, 15–21.

Fick, A.L., Feldstein, V.A., Norton, M.E., Wassel Fyr, C., Caughey, A.B., and Machin, G.A. (2006). Unequal placental sharing and birth weight discordance in monochorionic diamniotic twins. Am. J. Obstet. Gynecol. *195*, 178–183.

Green, C.J., and Day, M.L. (2013). Insulin-like growth factor 1 acts as an autocrine factor to improve early embryogenesis in vitro. Int. J. Dev. Biol. *57*, 837–844.

Harper, L.M., Weis, M.A., Odibo, A.O., Roehl, K.A., Macones, G.A., and Cahill, A.G. (2013). Significance of growth discordance in appropriately grown twins. Am. J. Obstet. Gynecol. *208*, 393.e1–393.e5.

Harvey, M.B., and Kaye, P.L. (1992). IGF-2 stimulates growth and metabolism of early mouse embryos. Mech. Dev. *38*, 169–173.

Home, P., Saha, B., Ray, S., Dutta, D., Gunewardena, S., Yoo, B., Pal, A., Vivian, J.L., Larson, M., Petroff, M., et al. (2012). Altered subcellular localization of transcription factor TEAD4 regulates first mammalian cell lineage commitment. Proc. Natl. Acad. Sci. USA *109*, 7362–7367.

Johnson, M.H., and Ziomek, C.A. (1981). The foundation of two distinct cell lineages within the mouse morula. Cell *24*, 71–80.

Kimber, S.J., Sneddon, S.F., Bloor, D.J., El-Bareg, A.M., Hawkhead, J.A., Metcalfe, A.D., Houghton, F.D., Leese, H.J., Rutherford, A., Lieberman, B.A., and Brison, D.R. (2008). Expression of genes involved in early cell fate decisions in human embryos and their regulation by growth factors. Reproduction *135*, 635–647.

Knopman, J.M., Krey, L.C., Oh, C., Lee, J., McCaffrey, C., and Noyes, N. (2014). What makes them split? Identifying risk factors that lead to monozygotic twins after in vitro fertilization. Fertil. Steril. *102*, 82–89.

Lewi, L., Cannie, M., Blickstein, I., Jani, J., Huber, A., Hecher, K., Dymarkowski, S., Gratacós, E., Lewi, P., and Deprest, J. (2007). Placental sharing, birthweight discordance, and vascular anastomoses in monochorionic diamniotic twin placentas. Am. J. Obstet. Gynecol. *197*, 587.e1–587.e8.

Lewi, L., Jani, J., Blickstein, I., Huber, A., Gucciardo, L., Van Mieghem, T., Doné, E., Boes, A.S., Hecher, K., Gratacós, E., et al. (2008). The outcome of monochorionic diamniotic twin gestations in the era of invasive fetal therapy: a prospective cohort study. Am. J. Obstet. Gynecol. *199*, 514.e1–514.e8.

Lighten, A.D., Moore, G.E., Winston, R.M., and Hardy, K. (1998). Routine addition of human insulin-like growth factor-I ligand could benefit clinical in-vitro fertilization culture. Hum. Reprod. *13*, 3144–3150.

Lorthongpanich, C., Messerschmidt, D.M., Chan, S.W., Hong, W., Knowles, B.B., and Solter, D. (2013). Temporal reduction of LATS kinases in the early preimplantation embryo prevents ICM lineage differentiation. Genes Dev. *27*, 1441–1446.

Makarevich, A.V., and Markkula, M. (2002). Apoptosis and cell proliferation potential of bovine embryos stimulated with insulin-like growth factor I during in vitro maturation and culture. Biol. Reprod. *66*, 386–392.

Mehta, B.N., Chimote, N.M., Chimote, M.N., Chimote, N.N., and Nath, N.M. (2013). Follicular fluid insulin like growth factor-1 (FF IGF-1) is a biochemical marker of embryo quality and implantation rates in in vitro fertilization cycles. J. Hum. Reprod. Sci. *6*, 140–146.

Niakan, K.K., and Eggan, K. (2013). Analysis of human embryos from zygote to blastocyst reveals distinct gene expression patterns relative to the mouse. Dev. Biol. *375*, 54–64.

Nishioka, N., Inoue, K., Adachi, K., Kiyonari, H., Ota, M., Ralston, A., Yabuta, N., Hirahara, S., Stephenson, R.O., Ogonuki, N., et al. (2009). The Hippo signaling pathway components Lats and Yap pattern Tead4 activity to distinguish mouse trophectoderm from inner cell mass. Dev. Cell *16*, 398–410.

Noli, L., Dajani, Y., Capalbo, A., Bvumbe, J., Rienzi, L., Ubaldi, F.M., Ogilvie, C., Khalaf, Y., and Ilic, D. (2015). Developmental clock compromises human twin model created by embryo splitting. Hum. Reprod., dev252. http://dx.doi.org/10.1093/humrep/dev252.

Payne, D., Okuda, A., Wakatsuki, Y., Takeshita, C., Iwata, K., Shimura, T., Yumoto, K., Ueno, Y., Flaherty, S., and Mio, Y. (2007). Time-lapse recording identifies human blastocysts at risk of producing monzygotic twins. Hum. Reprod. *22* (*Suppl 1*), i9–i10.

Saeed, A.I., Sharov, V., White, J., Li, J., Liang, W., Bhagabati, N., Braisted, J., Klapa, M., Currier, T., Thiagarajan, M., et al. (2003). TM4: a free, open-source system for microarray data management and analysis. Biotechniques *34*, 374–378.

Spanos, S., Becker, D.L., Winston, R.M., and Hardy, K. (2000). Anti-apoptotic action of insulin-like growth factor-I during

human preimplantation embryo development. Biol. Reprod. *63*, 1413–1420.

Thieme, R., Ramin, N., Fischer, S., Püschel, B., Fischer, B., and Santos, A.N. (2012). Gastrulation in rabbit blastocysts depends on insulin and insulin-like-growth-factor 1. Mol. Cell. Endocrinol. *348*, 112–119.

Tocino, A., Blasco, V., Prados, N., Vargas, M.J., Requena, A., Pellicer, A., and Fernández-Sánchez, M. (2015). Monozygotic twinning after assisted reproductive technologies: a case report of asymmetric development and incidence during 19 years in an international group of in vitro fertilization clinics. Fertil. Steril. *103*, 1185–1189.

Togashi, K., Kumagai, J., Sato, E., Shirasawa, H., Shimoda, Y., Makino, K., Sato, W., Kumazawa, Y., Omori, Y., and Terada, Y. (2015). Dysfunction in gap junction intercellular communication induces aberrant behavior of the inner cell mass and frequent collapses of expanded blastocysts in mouse embryos. J. Assist. Reprod. Genet. *32*, 969–976.

Velazquez, M.A., Zaraza, J., Oropeza, A., Webb, R., and Niemann, H. (2009). The role of IGF1 in the in vivo production of bovine embryos from superovulated donors. Reproduction *137*, 161–180.

Velazquez, M.A., Hermann, D., Kues, W.A., and Niemann, H. (2011). Increased apoptosis in bovine blastocysts exposed to high levels of IGF1 is not associated with downregulation of the IGF1 receptor. Reproduction *141*, 91–103.

Vitthala, S., Gelbaya, T.A., Brison, D.R., Fitzgerald, C.T., and Nardo, L.G. (2009). The risk of monozygotic twins after assisted reproductive technology: a systematic review and meta-analysis. Hum. Reprod. Update *15*, 45–55.

Xue, Z., Huang, K., Cai, C., Cai, L., Jiang, C.Y., Feng, Y., Liu, Z., Zeng, Q., Cheng, L., Sun, Y.E., et al. (2013). Genetic programs in human and mouse early embryos revealed by single-cell RNA sequencing. Nature *500*, 593–597.

Yan, L., Yang, M., Guo, H., Yang, L., Wu, J., Li, R., Liu, P., Lian, Y., Zheng, X., Yan, J., et al. (2013). Single-cell RNA-Seq profiling of human preimplantation embryos and embryonic stem cells. Nat. Struct. Mol. Biol. *20*, 1131–1139.

Stem Cell Reports, Volume *5*

Supplemental Information

Discordant Growth of Monozygotic Twins Starts at the Blastocyst Stage: A Case Study

Laila Noli, Antonio Capalbo, Caroline Ogilvie, Yacoub Khalaf, and Dusko Ilic

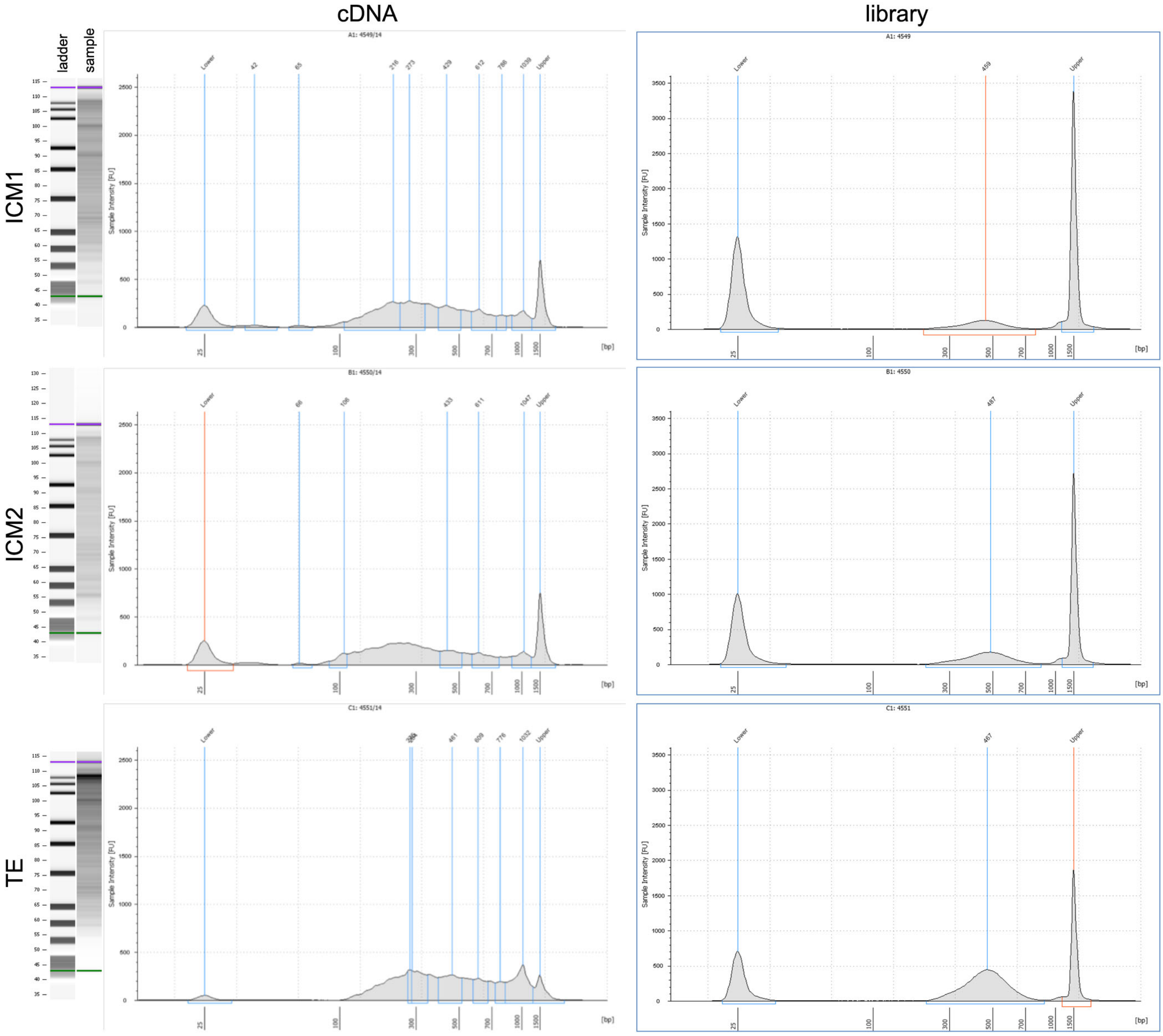

Figure S1. QC electropherograms. Size distribution profiles of cDNAs and libraries constructed from two ICM (ICM 1 and ICM2) and TE fraction of the monozygotic twin embryo.

A

Platform	Sample	Uniquely mapped reads	Properly paired
Illumina	ICM1	77.03%	74.32%
	ICM2	76.87%	74.53%
	TE	78.22%	76.76%
SOLiD	ICMP	80.64%	47.91%
	TEP	80.64%	48.40%

B

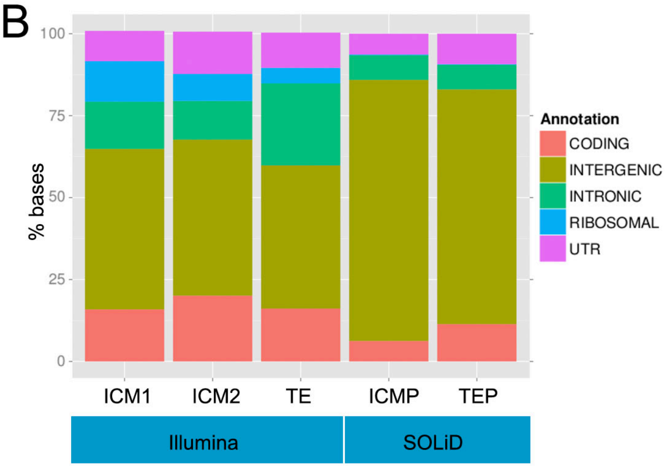

C

Platform	Sample	Ribosomal	Coding	UTR	Intronic	Intergenic	mRNA
Illumina	ICM1	12.35	15.89	9.26	14.42	48.96	25.15
	ICM2	8.25	20.08	12.90	11.82	47.60	32.98
	TE	4.74	16.13	10.76	25.02	43.70	26.89
SOLiD	ICMP	0.21	6.26	6.35	7.54	79.67	12.61
	TEP	0.16	11.40	9.32	7.53	71.61	20.72

D

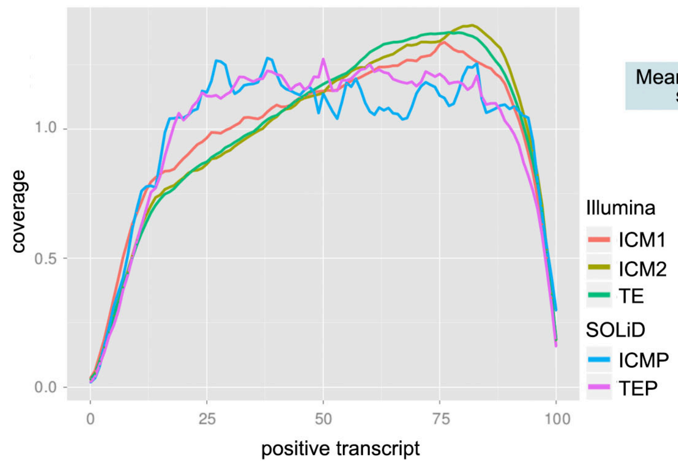

E

	Illumina			SOLiD	
	ICM1	ICM2	TE	ICMP	TEP
Mean insert size	67.9	79.4	72.5	40.7	44.5

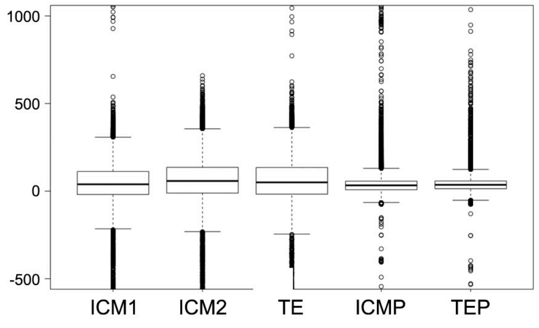

Figure S2. Quality control of mapping.

The samples are clustering by technology/batch rather than by biology. (A) Mapping statistics reveals high percentage of uniquely mapped reads on both platforms. Distribution of reads depending on genomic localization (B and C), the potential 5'/3' bias on transcript position (D), and estimated insert size (E) pointed to differences between two platforms.

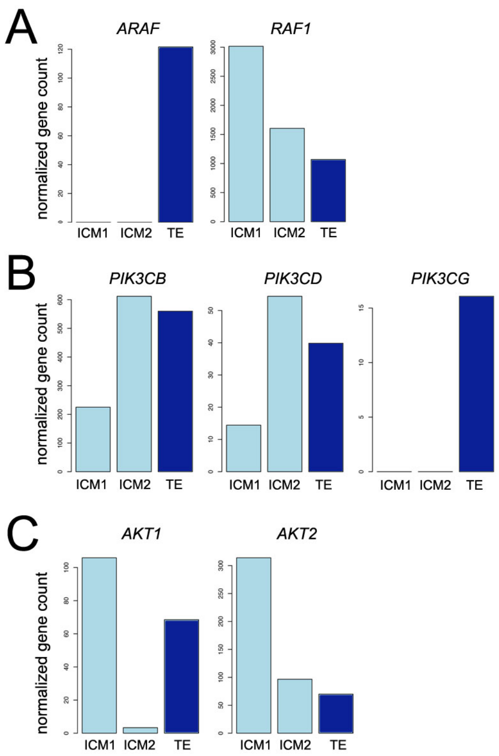

Figure S3. Expression levels of *RAF* (A), class I *PI3K* catalytic subunits (B) and *AKT* (C) gene family members that are not predominantly expressed in the ICM2.

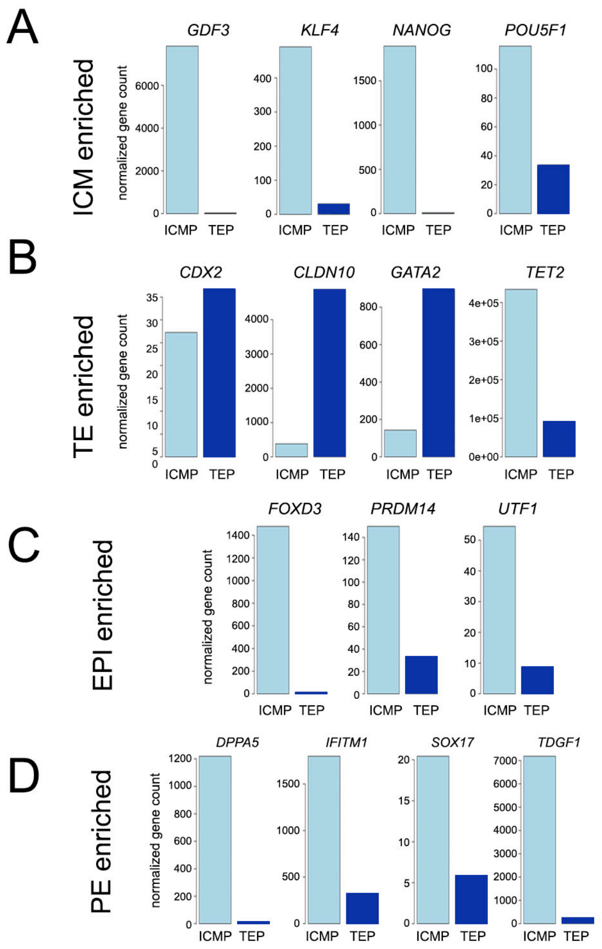

Figure S4. Lineage marker expression pattern confirms that the ICM samples are isolated form late blastocysts.

EPI, epiblast; ICM, inner cell mass; PE, primitive endoderm; TE, trophectoderm.
